# Designing chemical analogs to PbTe with intrinsic high band degeneracy and low lattice thermal conductivity

**DOI:** 10.1038/s41467-019-08542-1

**Published:** 2019-02-12

**Authors:** Jiangang He, Yi Xia, S. Shahab Naghavi, Vidvuds Ozoliņš, Chris Wolverton

**Affiliations:** 1 Department of Materials Science and Engineering, Northwestern University, Evanston, IL 60208 USA; 2 Department of Physical and Computational Chemistry, Shahid Beheshti University, G.C., Evin, Tehran, 1983969411 Iran; 3 Department of Applied Physics, Yale University, New Haven, CT 06520 USA; 4 Yale Energy Sciences Institute, West Haven, CT 06516 USA

## Abstract

High-efficiency thermoelectric materials require simultaneously high power factors and low thermal conductivities. Aligning band extrema to achieve high band degeneracy, as realized in PbTe, is one of the most efficient approaches to enhance power factor. However, this approach usually relies on band structure engineering, e.g., via chemical doping or strain. By employing first-principles methods with explicit computation of phonon and carrier lifetimes, here we show two full-Heusler compounds Li_2_TlBi and Li_2_InBi have exceptionally high power factors and low lattice thermal conductivities at room temperature. The expanded rock-salt sublattice of these compounds shifts the valence band maximum to the middle of the Σ line, increasing the band degeneracy by a factor of three. Meanwhile, resonant bonding in the PbTe-like sublattice and soft Tl–Bi (In–Bi) bonding interaction is responsible for intrinsic low lattice thermal conductivities. Our results present an alternative strategy of designing high performance thermoelectric materials.

## Abstract (web summary)

Finding new thermoelectric materials that can achieve high performance is vital to realizing the potential of future energy harvesting technology. Here, through computation methods, the authors identify two high-performance full-Heusler thermoelectric compounds with high intrinsic band degeneracy.

## Introduction

Thermoelectric (TE) materials have important applications in energy harvesting, thermoelectric coolers, and thermal detectors as they can directly convert heat into electricity and vise versa. Highly efficient TE materials are required for practical applications and are characterized by the figure of merit *zT* = (*S*^2^*σT*)/(*κ*_L_ + *κ*_e_), where *S*, *σ*, *κ*_e_, *κ*_L_, and *T* are the Seebeck coefficient, electrical conductivity, electronic thermal conductivity, lattice thermal conductivity, and temperature, respectively. In order to maximize *zT*, both electronic transport properties and lattice thermal conductivity have to be optimized carefully. Many strategies have been successfully used to suppress *κ*_L_^1^. However, there are fewer approaches that can effectively improve the electronic properties, i.e., the power factor (PF = *S*^2^*σ*)^2–4^. One effective route is to increase the band degeneracy (*N*_v_) and decrease the inertial effective mass $\left( {m_{\mathrm{I}}^ \ast } \right)$ simultaneously since the figure of merit *zT* of a material is proportional to $\frac{{N_{\mathrm{v}}}}{{m_{\mathrm{I}}^ \ast }}$^2,5^. Although a high density of states (DOS) effective mass ($m_{\mathrm{d}}^ \ast$ = $N_{\mathrm{v}}^{2/3}m_{\mathrm{b}}^ \ast$) is preferred for generating a high *S*^6,7^, the band effective mass $m_{\mathrm{b}}^ \ast$ is also concomitantly high in a material with low *N*_v_, leading to a low electrical conductivity as $\sigma \propto \frac{\tau }{{m_{\mathrm{b}}^ \ast }}$ (*τ* is the carrier lifetime)^5^.

A high value of *N*_v_ can be achieved either from a high valley multiplicity (the number of the carrier pockets of a band in the Brillouin zone) or a high orbital degeneracy (the number of bands with the same energy). Take the well studied TE material PbTe (rock-salt lattice, space group $Fm\bar 3m$) as an example, once the second maximum of the valence band (the middle of the Σ line, multiplicity is 12) is converged with the valence band maximum (VBM) (at the L point, multiplicity is 4) by alloying an appropriate amount PbSe, a significant enhancement of *zT* from 0.8 to 1.8 can be reached^8^. In practice, many materials have very limited dopability or the energy band can not be properly converged. Therefore, TE materials with intrinsically high band degeneracy are highly desired. Unfortunately, most intrinsic semiconductors have very low valley multiplicity. A high valley multiplicity usually only appears in cubic crystal systems where the VBM or conduction band minimum (CBM) is located at a low symmetry point of the first Brillouin zone, such as the Σ line of the rock-salt structure^2^. In addition to alloying, the band convergence could, in principle, be achieved through strain engineering. The lattice constant plays an important role on the alignment of Σ and L in PbTe^8,9^. However, a completely alignment requires an extremely large strain, which is not reachable in practice. Therefore, an alternative material design strategy is desired.

Semiconducting half-Heusler (HH) (chemical formula *XYZ*; space group $F\bar 43m$) compounds have been widely studied as TE materials due to their high power factors^10–12^ and excellent dopability^13,14^. Since semiconducting full-Heusler (FH) (chemical formula *X*_2_*YZ*; space group $Fm\bar 3m$) compounds are very rare, the study of FH TE is highly limited^15^. Owing to the structural similarity between HHs and FHs^16^, semiconducting FH compounds are expected to have good TE performance as well. The FH structure is a face centered cubic crystal structure with the interpenetration of *X*_2_ cubic and *YZ* rock-salt sublattices. The embedded cubic sublattice extends the bond length between *Y* and *Z* atoms of the rock-salt sublattice. Therefore, FH structure is an ideal candidate for realizing expanded PbTe.

In this work, one stable (Li_2_TlBi) and one metastable (Li_2_InBi) FH compounds with PbTe-like electronic structure, are discovered by combining a TE material design strategy and high throughput ab initio thermodynamic screening^15,17^, see Supplementary Note 1 for details. The crystal structure of FH Li_2_TlBi (Li_2_InBi) is the interpenetration of Li_2_ cubic and TlBi (In–Bi) rock-salt sublattices. The electronic structure of [Li^+^]_2_[Tl^+^Bi^3−^] ([Li^+^]_2_[In^+^Bi^3−^]) is isoelectronic with PbTe (Pb^2+^Te^2−^) since the electrons donated by two Li atoms are delocalized in the whole system. However, the bond length of Tl–Bi (In–Bi) is considerably extended in the FH lattice. Consequently, both the VBM and CBM of these two compounds are located in the middle of the Σ line, with band degeneracy of *N*_v_ = 12 in the intrinsic compounds due to their large lattice constants (~7.15 Å). Our transport calculations, explicitly including phonon–phonon and electron–phonon interactions, show that these two compounds have low *κ*_L_ and high PF at room temperature. Benefiting from their low *κ*_L_ and high PF, Li_2_InBi and Li_2_TlBi are therefore identified as promising room-temperature TE materials with ideal *zT* values of 1.5 and 2.0 at 300 K, respectively.

## Results

### Stability

**Fig. 1:**
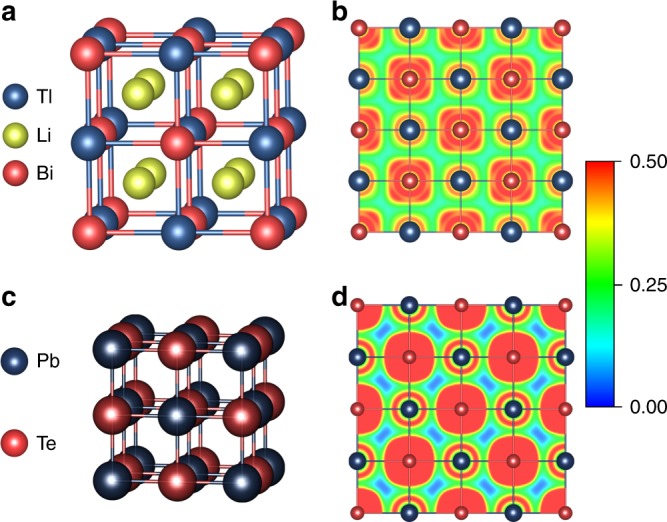
Crystal and electronic structures. **a** Crystal structure of full Heusler Li_2_TlBi. **b** Electron localization function (ELF) of Li_2_TlBi in (001) plane. **c** Crystal structure of PbTe. **d** ELF of PbTe in (001) plane

Our density functional theory (DFT) calculations show that FH Li_2_TlBi is on the *T* = 0 K convex hull, which means it is thermodynamically stable at zero Kelvin, and Li_2_InBi is just 4 meV/atom above the convex hull, which indicates it is thermodynamically weakly unstable (metastable). The convex hull distance (stability) is the formation energy difference between the target compound and its competing phases included in the Open Quantum Material Database (OQMD)^18^, which contains over 650,000 DFT calculations consisting of experimentally observed compounds from the international crystal structure database (ICSD)^19,20^ and hypothetical compounds with prototype structures. We also performed a ground state crystal structure search by using 21 distinct *X*_2_*YZ* prototype structures (see Supplementary Table 1) and the particle swarm optimization method as implemented in the CALYPSO code^21,22^, and we find the FH structure (see Fig. 1) is the lowest energy structure for both Li_2_InBi and Li_2_TlBi. Phonon calculations show that both Li_2_TlBi and Li_2_InBi are dynamically stable at *T* = 0 K. Free energy differences (Δ*G* = Δ*H* − *T*Δ*S*, where Δ*H* and Δ*S* are formation energy and formation entropy, respectively, see Supplementary Table 2 for details.) between FH phases and their competing phases in the corresponding ternary phase space show that Li_2_TlBi is thermodynamically stable and Li_2_InBi is slight unstable (~4 meV/atom above the convex hull) up to 500 K, see Supplementary Fig. 1. We count Li_2_InBi as synthesizable compound since such small convex hull distance is well within the threshold of Heusler compounds^23,24^.

### Electronic structure

**Fig. 2:**
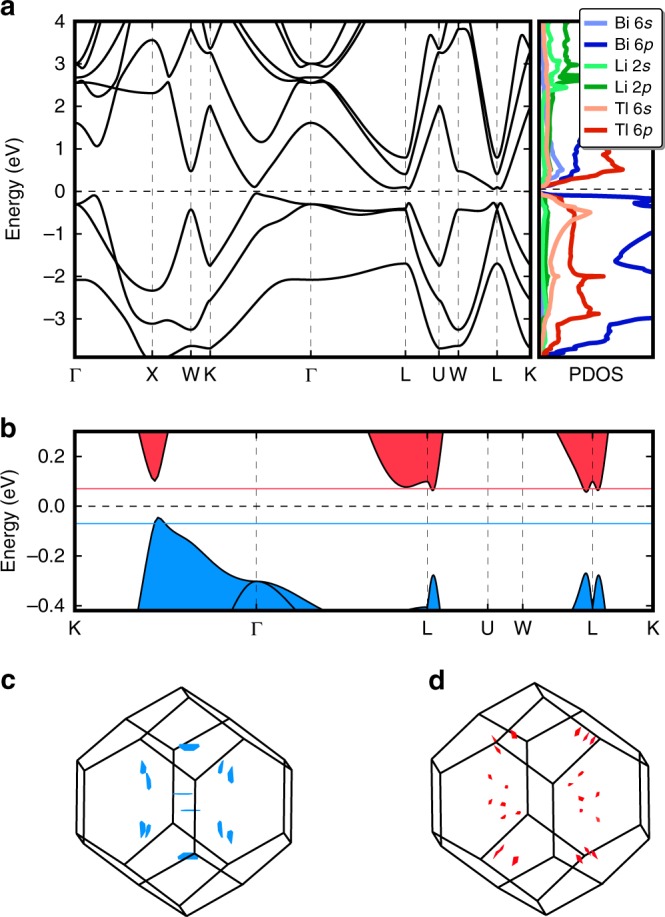
Electronic structure of Li_2_TlBi. **a** Band structure (left) and density of states (right) of Li_2_TlBi. **b** Expanded view of the band structure around the Fermi level. **c** Fermi surface of hole doped Li_2_TlBi. **d** Fermi surface of electron doped Li_2_TlBi. Note the size of Fermi pockets in electron doped Li_2_TlBi are re-scaled by a factor of two for the purpose of visualization. The Fermi level in the band structures is shifted to zero

**Fig. 3:**
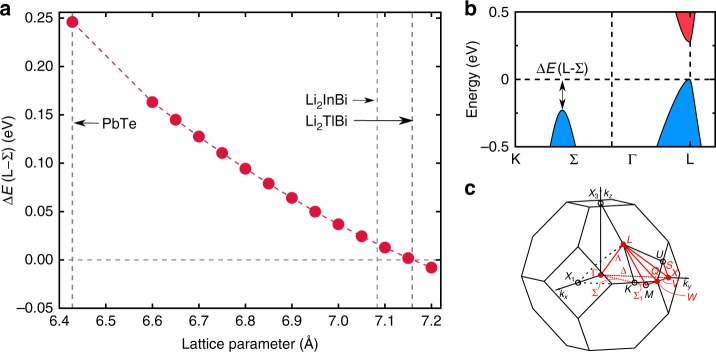
Lattice induced band convergence. **a** The valence band energy difference between L and Σ of PbTe as a function of lattice constant. The vertical lines indicates the lattice constants of experimental PbTe and fully relaxed Li_2_TlBi and Li_2_InBi. **b** Band structure (blue: valence band; red: conduction band) of PbTe along K-Γ-L direction. **c** High symmetry points/line of the first Brillouin zone of $Fm\bar 3m$

The main features of the Li_2_*Y*Bi (*Y* = In and Tl) band structure are determined by [*Y*^+^Bi^3−^]^2−^, which is isoelectronic with Pb^2+^Te^2−^ even though In/Tl (Bi) is cubic-coordinated with eight Li atoms as the nearest neighbors and octahedrally coordinated with six Bi (In/Tl) as the next nearest neighbor. This is because Li is the most electropositive element in these compounds and it donates its 2*s* electron to the crystal system. As shown in Fig. 2, Li 2*s* electron is completely delocalized in these compounds and therefore it has very limited influence on the electronic structures of Li_2_*Y*Bi (*Y* = In and Tl) except for raising the Fermi level and opening the band gap, which is similar to the Li stabilized quaternary Heusler semiconductors^16^. We further verified this conclusion by performing band structure calculations for [TlBi]^2−^ with the −2 charge balanced by a +2 Jellium background, see Supplementary Fig. 2. Since Tl is the nearest neighbor of Pb and Bi is close to Te in the periodic table, Li_2_InBi and Li_2_TlBi have very similar band structures with PbTe, as shown in Fig. 2a. Interestingly, Li_2_InBi and Li_2_TlBi have much larger lattice constants than PbTe because of the inserted Li_2_ cubic sublattice, which plays an important role in raising the energy level of the VBM at the middle of Σ line. As a consequence, the *N*_v_ of Li_2_*Y*Bi reaches to 12, as observed in the PbTe under significant hydrostatic expansion. As depicted in Fig. 3, a remarkable decrease in the energy difference between Σ and L is observed when the lattice constant of PbTe is expanded to that of Li_2_TlBi (Li_2_InBi). At the same time, the large bonding distance (softer bonding interaction) between Bi and *Y* (*Y* = In and Tl) contributes to reducing *κ*_L_ as we will see later^5^.

Since Li_2_InBi has a very similar electronic structure to Li_2_TlBi, we only take Li_2_TlBi as an example here. The electronic structure of Li_2_TlBi is shown in Fig. 2 (the band structure of Li_2_InBi is shown in Supplementary Fig. 3). Li_2_TlBi is a small band gap semiconductor (PBE: 0.06 eV; HSE06: 0.18 eV, including the spin–orbit coupling (SOC), which is consistent with a previous calculation^25^. These calculated gaps are well comparable with many high *zT* TE materials, such as PbTe: 0.19 eV^26^ and CoSb_3_: 0.05 ~ 0.22 eV^27,28^). In Li_2_TlBi, the band gap opens between the fully occupied Bi 6*p* and fully unoccupied Tl 6*p* states due to charge transfer from Tl to Bi. Tl atom loses its one 6*p* electron to the more electronegative Bi atom and becomes Tl^+^, and its 6*s*^2^ electrons are deeply (~−5 eV below the Fermi level) buried below the Bi 6*p* orbitals (valence bands, from −4 to 0 eV), forming stereochemically inactive lone-pair electrons. Two electropositive Li atoms lose their 2*s* electrons to Bi as well. Therefore, the 6*p* orbitals of Bi^3−^ (from −4 to 0 eV below the Fermi level) are fully filled with six electrons. The splitting of three occupied Bi 6*p* orbitals into two groups, ~−2 eV (single degeneracy) and ~−0.5 eV (double degeneracy) at the Γ point is due to SOC. The conduction bands are mainly from the Tl^+^ 6*p* orbitals. The electron localization function (ELF) is shown in Fig. 1 and Supplementary Fig. 4. We can clearly see that Bi and Li atoms have, respectively, the highest and lowest ELF values, consistent with our electronic structure analysis that Li donates its electrons to the system while Bi gains electrons. The inactive lone-pair electrons of Tl^+^ 6*s*^2^ are clearly observed in Fig. 1 as well, which is analogous to Pb^2+^ in PbTe. Finally, the ELF of Li_2_TlBi is very similar to that of PbTe, which is identified as a typical resonant bonding system^29,30^.

As expected from the previous analysis, a remarkable feature of the Li_2_TlBi band structure is that its VBM lies in the middle of Σ line of the first Brillouin zone of the FCC FH structure $\left( {Fm\bar 3m} \right)$, which leads to an unexpected high valley degeneracy (*N*_v_ = 12), see the Fermi surface in Fig. 2. Hence the *N*_v_ = 12 of the VBM reaches a record high value, which only has been previously matched in the heavily doped PbTe and CoSb_3_ systems^8,31^. The second hole pocket, which is ~40 meV lower than VBM, is located at the middle of the Δ line (between Γ and X) and possesses a valley degeneracy of 6. Therefore, an extremely high *N*_v_ = 18 is reachable in Li_2_TlBi by means of hole doping. Although the CBM is located at L with the valley degeneracy of 4, the energy difference between CBM and the second highest electron pocket (in the middle of the Σ line) is only 7 meV. Therefore the *N*_v_ of the conduction band can potentially reach as high as 16 through light electron doping. The Fermi surfaces of the valence and conduction bands are displayed in Fig. 2. As mentioned above, although the band effective masses $\left( {m_{\mathrm{b}}^ \ast } \right)$ for the VBM and CBM are small, which imply high carrier mobilities as $\mu \propto \frac{\tau }{{m_{\mathrm{b}}^ \ast }}$, the Seebeck coefficient $S \propto m_{\mathrm{d}}^ \ast$ still can be very high, provided *N*_v_ is sufficiently large, since $m_{\mathrm{d}}^ \ast$ is related to the band effective mass by $m_{\mathrm{d}}^ \ast = N_{\mathrm{v}}^{2/3}m_{\mathrm{b}}^ \ast$.

### Electron transport

**Fig. 4:**
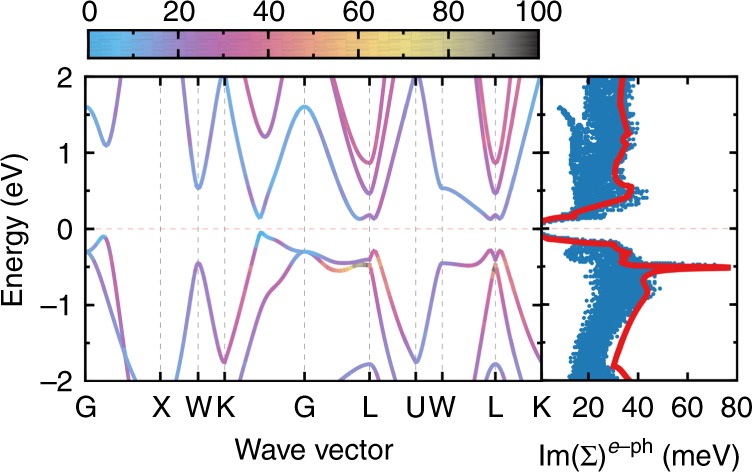
Electron–phonon coupling. Heat map of the imaginary part of the electron–phonon self-energy (Im(Σ)) of Li_2_TlBi at 300 K (left) and mode-dependent Im(Σ) compared with scaled density of states (DOS) (right). Fermi level is shifted to zero

To quantitatively characterize the electron transport properties of Li_2_*Y*Bi (*Y* = In and Tl), we calculate *S* and *σ* based on the semiclassical Boltzmann transport equation under relaxation time approximation. We assume that the predominant carrier scattering mechanisms at 200 K and above are all based on phonons: (1) deformation potentials of acoustic and optical phonons and (2) Fröhlich coupling due to polar optical phonons^32,33^. Since the best thermoelectric efficiency is always achieved in the heavily doped region where the scattering on polar optical phonons is sufficiently screened and the dielectric constant is usually large in narrow band gap semiconductors^32–34^, we mainly take into account deformation potential scattering using first-principles calculated electron–phonon interaction (EPI) matrix elements. As shown in Fig. 4 for the representative compound Li_2_TlBi, the imaginary part of the electron self-energy Im(Σ) shows a strong energy dependence and is roughly proportional to the density of electronic states. States with a long lifetime appear near the VBM and CBM. This indicates that the lifetime is linked to the phase space availability for electronic transitions, i.e., electrons and holes near the band edges are less scattered due to limited phase space^35^.

**Fig. 5:**
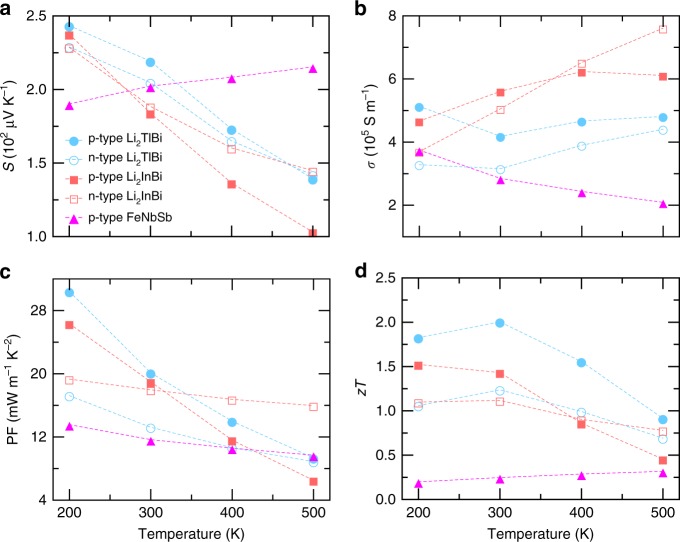
Electron transport properties. **a**–**d** The Seebeck coefficient *S* (**a**), electrical conductivity *σ* (**b**), power factor PF (**c**), and *zT* (**d**) of Li_2_TlBi and Li_2_InBi at carrier concentrations that give rise to maximum *zT* at 200, 300, 400, and 500 K, compared with p-type FeNbSb. The lattice thermal conductivity of FeNbSb used to compute *zT* is extracted from ref. ^36^. The electronic thermal conductivity (*κ*_e_) is shown in Supplementary Figs. 4 and 5

To validate our calculations, we also computed the thermoelectric properties for a well studied p-type HH compound FeNbSb, for which a PF as large as 10.6 mW m^−1^ K^−2^ was recently measured at room temperature^36^. Figure 5c shows that our calculation considering electron–phonon coupling predicts a maximum PF of 11.7 mW m^−1^ K^−2^ for FeNbSb at 300 K, representing the upper limit without considering other scattering sources such as defects and grain boundaries. Our calculated *S*, *σ*, and PF of FeNbSb at optimized carrier concentration and temperatures from 200 to 500 K also compare well with a recent theoretical study that employs the same methodology^37^. The good agreement between our calculations and experimental data confirms our assumption that electron–phonon coupling dominates carrier scattering in this system. It is noteworthy that the optimal PF of FeNbSb is significantly higher than that of PbTe at 300 K^8,38,39^.

Next, we illustrate the ultrahigh PFs of Li_2_TlBi and Li_2_InBi by comparing to FeNbSb. Despite the fact that *S* is generally much higher in FeNbSb (see Supplementary Figs. 5 and 6) due to its larger band gap of 0.54 eV compared to 0.18 eV (Li_2_TlBi) and 0.15 eV (Li_2_InBi), the *S* of Li_2_TlBi and Li_2_InBi is comparable with that of FeNbSb at optimal carrier concentration, particularly at 300 K, as shown in Fig. 5a. The strong bipolar effect further suppresses *S* of Li_2_TlBi and Li_2_InBi at higher temperatures. However, owing to the smaller band effective mass $\left( {m_{\mathrm{b}}^ \ast } \right)$ and high valley degeneracy (*N*_v_), both Li_2_TlBi and Li_2_InBi have significantly higher *σ* than FeNbSb from 300 to 500 K with a carrier concentration about one order of magnitudue lower than FeNbSb (see Supplementary Figs. 5 and 6). As a consequence, Li_2_TlBi (Li_2_InBi) achieves exceptional PFs of 30.4/20.1 (26.3/19.0) mW m^−1^ K^−2^ at 200/300 K, nearly twice that of FeNbSb at 300 K. The outperformance of Li_2_TlBi and Li_2_InBi over FeNbSb is due to a comparable *S* and a higher *σ* at the optimized carrier concentrations, supporting our previous discussion.

### Phonon transport

**Fig. 6:**
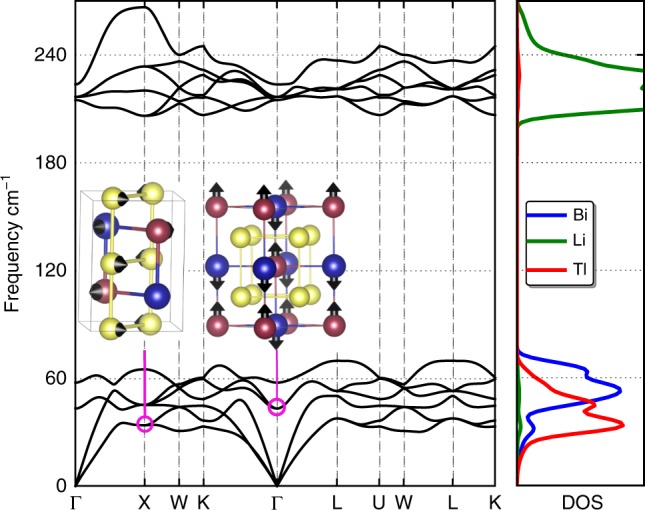
Phonon dispersion. Phonon spectra (left) and phonon density of states (right) of Li_2_TlBi. The longitudinal optical (LO) and transverse optical (TO) splitting is included. Insets are the phonon vibration models in real space at the *k*-point and energy indicated by pink circle

The Li_2_TlBi (Li_2_InBi) primitive cell contains four atoms and therefore 12 phonon branches. The mode decomposition in the zone center (Γ point) is 3T_1*u*_ ⊕ 1T_2*g*_. As shown in Fig. 6, the low-frequency phonon modes are mainly from the stereochemically inert lone-pair Tl^+^ cation instead of the heaviest atom Bi, which is consistent with the weaker bonding between Tl atom and its neighbors. As expected, the light lithium atom has much higher phonon frequencies 200 ~ 250 cm^−1^ and its phonon bands are completely separated from Tl and Bi. It is worth noting that these compounds possess two main differences from the previously reported alkali metal based rattling (*R*) Heusler^15^: (i) higher acoustic phonon frequencies, and (ii) higher frequency of crossing bands between acoustic and optical modes, meaning Tl (In) atom has a slightly stronger interaction with its neighbors than *R*-Heusler compounds.

**Fig. 7:**
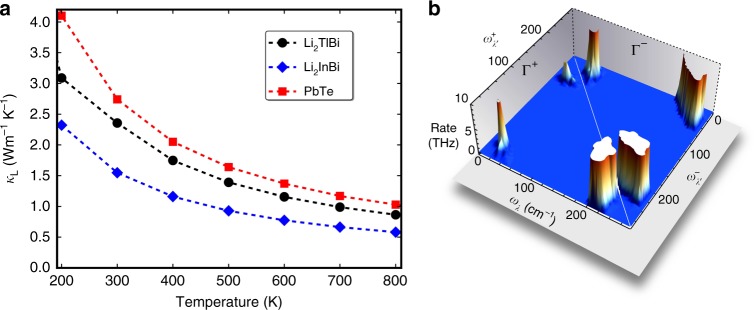
Phonon transport properties. **a** Calculated lattice thermal conductivity *κ*_L_ of Li_2_TlBi, Li_2_InBi, and PbTe as function of temperature. **b** Phonon scattering rates of Li_2_TlBi in absorption (Γ^+^: *λ* + *λ*′ → *λ*″) and emission (Γ^−^: *λ*″ → *λ* + *λ*′) processes

The lattice thermal conductivity *κ*_L_ is calculated by using first-principles compressive sensing lattice dynamics (CSLD) and solving the linear Boltzmann transport equation (see Methods for details) and the results are shown in Fig. 7. Owing to the cubic symmetry, *κ*_L_ of Li_2_TlBi and Li_2_InBi are isotropic ($\kappa _{\mathrm{L}}^{xx}$ = $\kappa _{\mathrm{L}}^{yy}$ = $\kappa _{\mathrm{L}}^{zz}$ = *κ*_L_) and the calculated *κ*_L_ are 2.36 (1.55) Wm^−1^ K^−1^ at 300 K and 0.55 (0.52) Wm^−1^ K^−1^ at 900 K for Li_2_TlBi (Li_2_InBi), which are much lower than most FH and HH (≥7 Wm^−1^ K^−1^^40^) compounds without doping or nanostructuring and also lower than PbTe (2.74 at 300 K and 0.91 Wm^−1^ K^−1^ at 900 K at the same computational level).

Similar to PbTe, Li_2_InBi, and Li_2_TlBi have low-lying transverse optical modes (TO), as shown in Supplementary Figs. 7 and 8, further confirming the presence of the resonant bonding^29^, as expected from earlier electronic structures analysis. The long-range interaction caused by the resonant bonding leads to strong anharmonic scattering and large phase space for three-phonon scattering processes and, therefore, significantly suppresses lattice thermal transport^29^. Moreover, the weak Tl–Bi (In–Bi) bonding resulting from the large bonding distance between Tl and Bi (In and Bi) gives rise to low group velocities. Finally, the high-frequency optical modes associated with the Li atoms provide extra scattering channels for low-lying acoustic modes.

The mechanism of the strong scattering of heat carrying acoustic phonon modes can be directly understood from phonon–phonon interactions. We show the phonon–phonon scattering rates in the absorption (Γ^+^: *λ* + *λ*′ → *λ*″) and emission (Γ^−^: *λ*″ → *λ* + *λ*′) processes in Fig. 7. The low-frequency acoustic phonons are mainly scattered by the low-frequency optical modes in the absorption process, while the optical modes decompose largely into low energy acoustic modes in the emission process. This scattering picture is similar to the alkali metal based *R*-Heusler compounds^15^.

## Discussion

Using our calculated *κ*_L_, *S*, *σ*, and *κ*_e_ within DFT framework by explicitly including electron–phonon and phonon–phonon interactions, the maximum figure of merit *zT* of Li_2_TlBi and Li_2_InBi are estimated to be 2.0 and 1.4 at 300 K for hole doping (p-type), respectively, which implies that Li_2_TlBi is the TE material with the highest *zT* at room temperature. Note that our calculated *κ*_L_ might be overestimated due to neglect of the phonon–phonon interaction beyond the third-order and phonon scattering by defects and grain boundaries. Furthermore, the *zT* of these FH materials could be further enhanced by suppressing heat transport through nanostructuring precipitates and optimizing grain size as commonly used in other Heusler compounds if the optimized carrier concentration can still be achieved. The optimized hole concentrations within the rigid band approximation for the maximum *zT* at room temperature are 1.3 × 10^19^ and 1.6 × 10^19^ cm^−3^, respectively, for Li_2_TlBi and Li_2_InBi (see Supplementary Figs. 5 and 6), which is close to those for PbTe at room temperature^38,39^ but one order of magnitude lower than in FeNbSb^36^. Since Li_2_TlBi has a better electronic structure than PbTe, the maximum *zT* at the same carrier concentration and room temperature is also higher^39^.

For experiments, it is important to ascertain thermodynamic limit to the achievable dopant concentration. To estimate the defect solubility of forming p-type semiconductor, neutral defects of Li and Tl vacancy were considered. The calculated vacancy formation energy (*E*_d_) of Li (Tl) in Li_2_TlBi is 0.29 (0.31) eV per vacancy in Li (Tl) poor condition. These values are comparable with that of the Na doped PbTe (0.27 eV per defect)^41^, where the hole concentration can reach 10^20^ cm^−3^ at room temperature^42,43^. These values strongly suggest that the required hole concentrations for maximizing *zT* are achievable in Li_2_TlBi.

Owing to the small band gap, the maximum *zT* values of Li_2_TlBi and Li_2_InBi are at room temperature, see Fig. 5. The drop down of the *zT* at higher temperature is mainly due to the decreased PF by the bipolar effect, stemming from their small band gaps since we assume that the band gap does not change with temperature. If their band gaps widen with elevated temperature as observed in PbTe, the maximum *zT* will be achieved at higher temperature. We also note that the electron doped (n-type) Li_2_TlBi and Li_2_InBi have high *zT* at room temperature as well, due to the high conduction band degeneracy (at Σ line and L point) and low lattice thermal conductivity. A material with high *zT* for both hole and electron doping is important for fabricating TE devices. Therefore, Li_2_TlBi and Li_2_InBi are promising materials for room temperature thermoelectric applications.

In summary, we discover two promising room-temperature TE materials, Li_2_TlBi and Li_2_InBi FHs, by high throughput stability screening and TE material design strategy of creating the analogs with isovalent electronic structures to PbTe with much expanded lattices. We demonstrate that Li_2_TlBi and Li_2_InBi possess intrinsic high PFs and low *κ*_L_ by using the state-of-the-art computational methods which combines the electron Boltzmann transport theory with ab initio carrier relaxation-time from electron–phonon coupling and phonon transport theory with phonon lifetime from first-principles CSLD. The high TE performance of the p-type Li_2_TlBi and Li_2_InBi at room temperature are mainly due to the high *N*_v_ induced by the extended lattice and the low *κ*_L_ caused by the resonant bonding as observed in PbTe and weak bonding interactions of the extended lattice, respectively. Our TE material design strategy enhances the band degeneracy and suppresses lattice thermal conductivity of the PbTe-type materials simultaneously. It is also straightforward to be extended to design or discover other TE materials.

## Methods

### DFT calculation details

In this study, most DFT calculations are performed using the Vienna Ab initio Simulation Package (VASP)^44,45^. The projector augmented wave (PAW^46,47^) pseudo potential, plane wave basis set, and Perdew–Burke–Ernzerhof (PBE^48^) exchange-correlation functional were used. The qmpy^18^ framework and the Open Quantum Material Database (OQMD)^18^ was used for convex hull construction. The band gap was computed by means of the screened hybrid functional HSE06^49^, including spin–orbit coupling (SOC).

### Crystal structure prediction

The lowest energy structure of Li_2_*Y*Bi were confirmed by prototype structure screening^15^ and crystal structure searching using the particle swarm optimization method as implemented in the CALYPSO code^21,22^.

### Free energy calculations

The lattice dynamic stability and vibrational entropy were computed by performing frozen phonon calculation as implemented in phonopy package^50^. The disordered competing phases were simulated using the special quasirandom structures (SQS) as implemented in the alloy theoretic automated toolkit (ATAT)^51^. Vibrational entropies of the ordered phases and SQSs of the disordered phases are calculated by using phonopy package^50^. Configuration entropy is included if the competing phases have atom disorder. The calculated results are shown in the Supplementary Table 2.

### Phonon and electron transport calculations

The CSLD^52^ technique was employed to obtain the third-order force constants, which were used to iteratively solve the linearized phonon Boltzmann transport equation with the ShengBTE package^53^. The carrier lifetime due to electron–phonon coupling was computed by using Quantum Espresso and Electron–phonon Wannier (EPW) codes with SOC included^35,54–56^. Thermoelectric properties including Seebeck coefficient (*S*), electrical conductivity (*σ*), and electronic thermal conductivity (*κ*_e_) were computed using BoltzTrap code^57^ with the adjusted band gap from HSE06 calculations and mode-resolved carrier lifetime from EPW.

## Supplementary information

Supplementary Information

## Data Availability

All data are available from the corresponding authors upon reasonable request. All codes used in this work are either publicly available or available from the authors upon reasonable request.
