# Supplementary material for: Designing chemical analogs to PbTe with intrinsic high band degeneracy and low lattice thermal conductivity
Source: Nat Commun. 2019 Feb 12;10:719. doi: 10.1038/s41467-019-08542-1 (PMC6372688; doi:10.1038/s41467-019-08542-1)
Supplement: Supplementary file 1 — Supplementary Information [file 41467_2019_8542_MOESM1_ESM.pdf]

Supplementary Information: Designing Chemical Analogs to PbTe with Intrinsic High Band Degeneracy and Low Lattice Thermal Conductivity

Jiangang He[1], Yi Xia[1], S. Shahab Naghavi[2,1], Vidvuds Ozoliņš[3,4], Chris Wolverton[1*]

[1] *Department of Materials Science and Engineering, Northwestern University, Evanston, IL 60208, USA*

[2] *Department of Chemistry, Shahid Beheshti University, Evin, 1983963113 Tehran, Iran*

[3] *Department of Applied Physics, Yale University, New Haven, CT, 06520, USA*

[4] *Yale Energy Sciences Institute, West Haven, CT, 06516, USA*

Email: c-wolverton@northwestern.edu

November 28, 2018

Contents

- Supplementary Figures:
  - Supplementary Figure 1: Formation free energy as a function of temperature for Li_2TlBi and Li_2InBi
  - Supplementary Figure 2: Band structure of $[TlBi]^{2-}$ with Li_2TlBi lattice constant
  - Supplementary Figure 3: Band structure of Li_2InBi
  - Supplementary Figure 4: Electron localization function of Li_2TlBi, PbTe, and NaCl
  - Supplementary Figure 5: Electron transport properties of Li_2TlBi
  - Supplementary Figure 6: Electron transport properties of Li_2InBi
  - Supplementary Figure 7: Phonon band structure and density of states of PbTe
  - Supplementary Figure 8: Phonon band structure and density of states of Li_2InBi
- Supplementary Tables:
  - Supplementary Table 1: Prototype structures used for crystal structure determination
  - SupplementaryTable 2: Energies and entropy of the phases in Li-Tl-Bi and Li-In-Bi chemical space
- Supplementary Notes
  - Supplementary note 1: High throughput thermal stability screening processing
- Supplementary References

Supplementary Figures

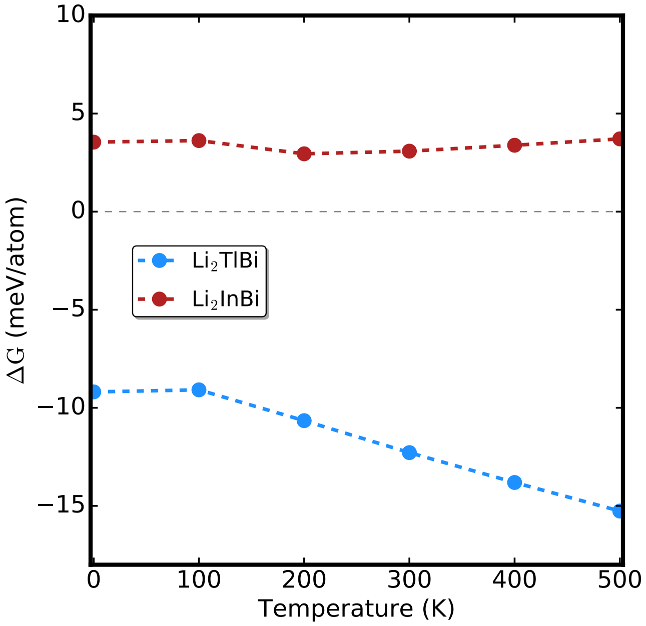

Supplementary Figure 1: Free energy difference ($\Delta G = \Delta H - T\Delta S$, where ΔH and ΔS are formation energy and formation entropy, respectively) between Li_2TlBi (Li_2InBi) and its competing phases in Li-Tl(In)-Bi phase space as a function of temperature. Negative (positive) value means the Heusler compound is stable (unstable).

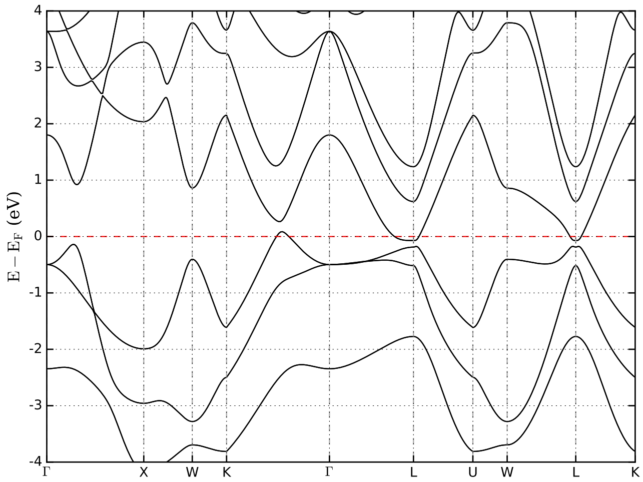

Supplementary Figure 2: Band structure of $[TlBi]^{2-}$ with Li_2TlBi lattice constant. The charge is balanced by a +2 Jellium background.

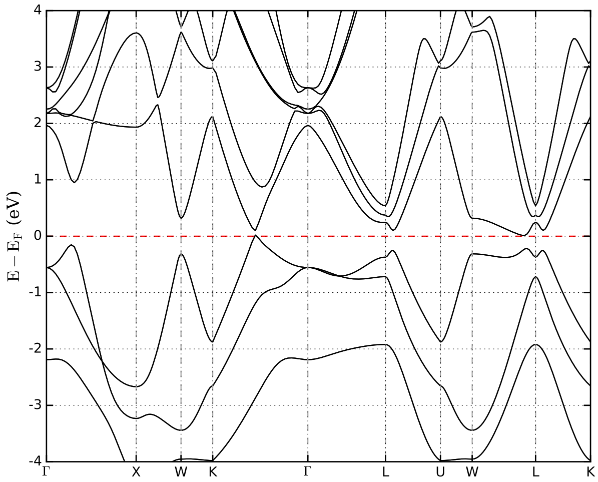

Supplementary Figure 3: Band structure of Li_2InBi computed by using PBE, including SOC.

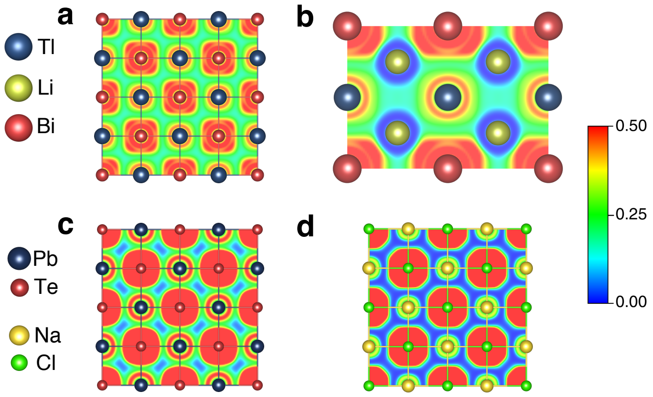

Supplementary Figure 4: (a) and (b) are the electron localization function (ELF) of Li_2TlBi along (001) and (110) planes, respectively. (c) the ELF of PbTe along (001) plane. (d) the ELF of NaCl along (001) plane.

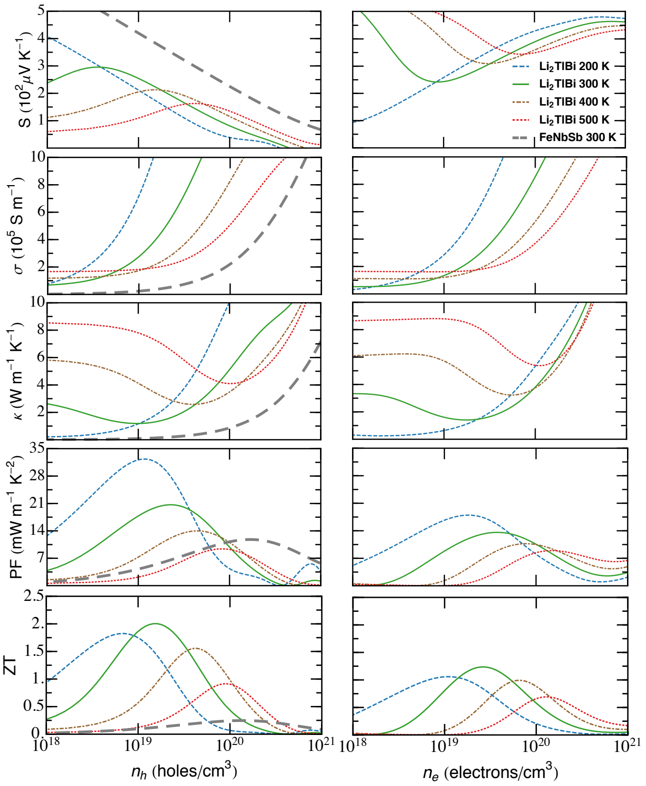

Supplementary Figure 5: The Seebeck coefficient (S), electronic conductivity (σ), electron thermal conductivity (κ_e), Power factor (PF, σS^2), and zT of Li_2TlBi as functions of carrier concentrations at 200, 300, 400 and 500 K, comparing with p-type FeNbSb. The lattice thermal conductivity of FeNbSb used to compute zT is extracted from Ref. 2.

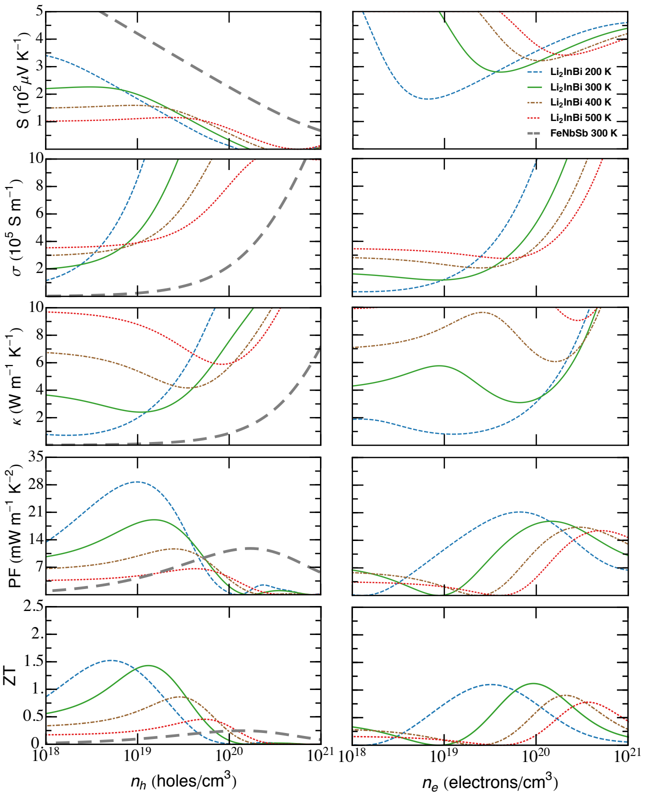

Supplementary Figure 6: The Seebeck coefficient (S), electronic conductivity (σ), electron thermal conductivity (κ_e), Power factor (PF, σS^2), and zT of Li_2InBi as functions of carrier concentrations at 200, 300, 400 and 500 K, comparing with p-type FeNbSb. The lattice thermal conductivity of FeNbSb used to compute zT is extracted from Ref. 2.

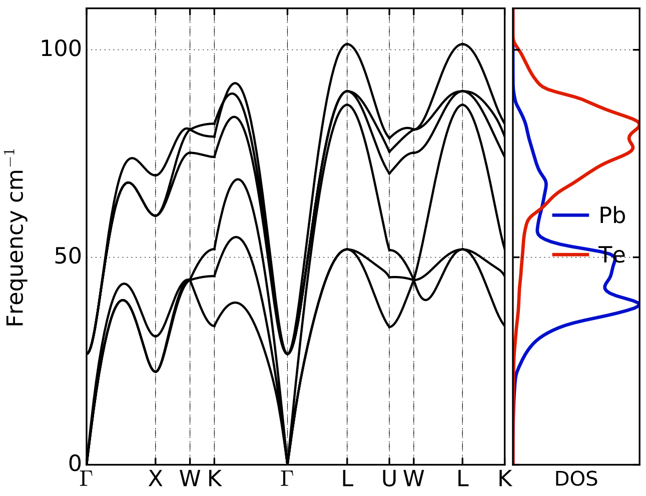

Supplementary Figure 7: Phonon band and DOS of PbTe.

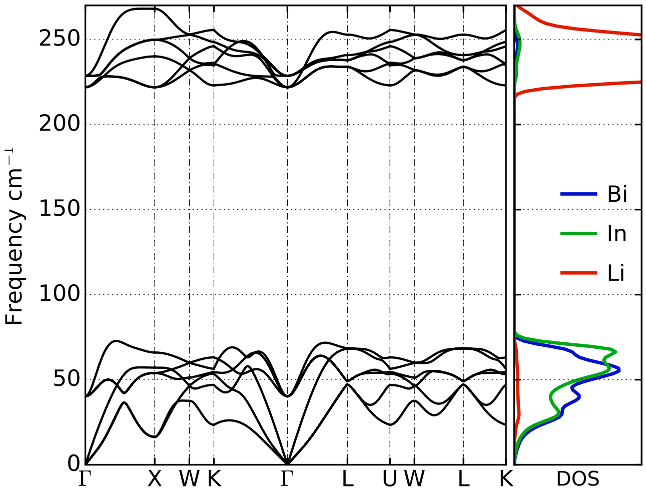

Supplementary Figure 8: Phonon band and DOS of Li_2InBi.

Supplementary Tables

Supplementary Table 1: The prototype crystal structures (space group, formula unit per primitive cell Z, and the number of intermetallic compound in Pearson's database, http://www.crystalimpact.com/pcd/) used in crystal structure determination.

	Prototype structure	space group	Z	Number of intermetallic compound in Pearson's database[1]
1	Cu_2MnAl	$Fm\bar{3}m$	4	333
2	Si_2CeNi	$Cmcm$	8	187
3	Si_2CuHf	$P4/nmm$	8	105
4	Al_2MgCu	$Cmcm$	8	81
5	Li_2AgSb	$F\bar{4}3m$	4	64
6	Ge_2YIr	$Immm$	32	35
7	Si_2CrZr	$Pbam$	48	31
8	Cu_3Ti (Ni_2TiCu)	$Pmmn$	8	23
9	Pd_2YSi	$Pmna$	16	17
10	Rh_2SnV	$I4/mmm$	8	13
11	Si_2LiCa	$Pnma$	16	9
12	Ga_2NdNi	$Cmmm$	16	8
13	Na_2CuAs	$Cmcm$	8	5
14	Pd_2USn	$Pnma$	16	4
15	Co_2NiGa	$P4/mmm$	4	3
16	Li_2CuAs	$P6_3/mmc$	8	3
17	Ni_2MnGa	$Pnnm$	24	1
18	Pt_2NiGe	$Fmmm$	8	1
19	Li_2ZnSi	$P\bar{3}m1$	8	1
20	Pt_2CdZn	$P4/mmm$	4	1
21	Al_2CuIr	$Cmme$	8	1
22	Pt_2CdZn	$P4/mmm$	4	1

Supplementary Table 2: Total energies (eV/f.u.) and entropies (J/K/mol/f.u.) of the compounds in Li-Tl-Bi and Li-In-Bi chemical space. S_{vib} and S_{conf} are vibrational and configuration entropy, respectively.

Comp.	Structure	Energy	$S_{\mathrm{vib}}^{100\mathrm{K}}$	$S_{\mathrm{conf}}^{100\mathrm{K}}$	$S_{\mathrm{vib}}^{200\mathrm{K}}$	$S_{\mathrm{conf}}^{200\mathrm{K}}$	$S_{\mathrm{vib}}^{300\mathrm{K}}$	$S_{\mathrm{conf}}^{300\mathrm{K}}$	$S_{\mathrm{vib}}^{400\mathrm{K}}$	$S_{\mathrm{conf}}^{400\mathrm{K}}$	$S_{\mathrm{vib}}^{500\mathrm{K}}$	$S_{\mathrm{conf}}^{500\mathrm{K}}$
Li	$Im\bar{3}m$	-1.904	6.619	0.000	17.814	0.000	26.457	0.000	33.082	0.000	38.387	0.000
Tl	$P63/mmc$	-2.227	39.912	0.000	56.883	0.000	66.937	0.000	74.092	0.000	79.648	0.000
Bi	$R\bar{3}m$	-3.885	30.476	0.000	46.933	0.000	56.769	0.000	64.006	0.000	69.546	0.000
In	$I4/mmm$	-2.561	33.100	0.000	49.749	0.000	59.740	0.000	66.873	0.000	72.419	0.000
LiTl	$Pm\bar{3}m$	-4.592	41.541	0.000	68.658	0.000	87.138	0.000	100.843	0.000	111.668	0.000
LiBi	$P4/mmm$	-6.565	39.259	0.000	67.891	0.000	86.803	0.000	100.674	0.000	111.580	0.000
Li_2Bi	$P\bar{6}2m$	-9.188	40.101	0.000	77.191	0.000	103.904	0.000	124.186	0.000	140.254	0.000
Li_2Tl	$Cmcm$	-6.762	43.925	0.000	80.632	0.000	107.253	0.000	127.382	0.000	143.413	0.000
Li_3Bi	$Fm\bar{3}m$	-11.800	46.717	0.000	93.392	0.000	128.183	0.000	154.738	0.000	175.975	0.000
Li_3Tl	$Fm\bar{3}m$	-8.879	51.528	0.000	98.151	0.000	133.011	0.000	159.620	0.000	180.880	0.000
Li_5Tl_2	$R\bar{3}m$	-15.661	97.410	0.000	180.894	0.000	242.422	0.000	289.161	0.000	326.459	0.000
Tl_3Bi	$Cmcm$	-10.695	158.053	0.000	225.753	0.000	265.934	0.000	294.541	0.000	316.760	0.000
$TlBi_2$	$P6/mmm$	-10.049	105.351	0.000	155.766	0.000	185.780	0.000	207.165	0.000	223.779	0.000
TlBi	SQS	-6.087	79.168	5.763	112.971	5.763	133.052	5.763	147.352	5.763	158.460	5.763
Tl_4Bi	SQS	-12.811	200.330	4.161	285.153	4.161	335.419	4.161	371.191	4.161	398.971	4.161
Li_2TlBi	Fm$\bar{3}$m	-11.465	83.490	0.000	140.424	0.000	178.169	0.000	205.883	0.000	227.682	0.000
$Li_{13}In_3$	$Fd\bar{3}m$	-35.915	138.807	0.000	314.809	0.000	451.218	0.000	556.387	0.000	640.833	0.000
Li_2In	$Cmcm$	-7.277	32.568	0.000	67.531	0.000	93.657	0.000	113.591	0.000	129.528	0.000
Li_3In_2	$R\bar{3}m$	-12.392	59.578	0.000	120.448	0.000	164.743	0.000	198.261	0.000	224.966	0.000
LiIn	$Pm\bar{3}m$	-5.058	34.307	0.000	59.995	0.000	78.051	0.000	91.588	0.000	102.333	0.000
$LiIn_3$	$Pm\bar{3}m$	-10.182	105.812	0.000	168.160	0.000	207.197	0.000	235.390	0.000	257.416	0.000
Li_5In_4	$P\bar{3}m1$	-22.486	118.324	0.000	231.559	0.000	312.369	0.000	373.128	0.000	421.405	0.000
InBi	$P4/nmm$	-6.454	59.412	0.000	92.366	0.000	112.282	0.000	126.523	0.000	137.604	0.000
Li_3In	$Fm\bar{3}m$	-9.289	40.045	0.000	84.484	0.000	118.692	0.000	145.025	0.000	166.157	0.000
In_2Bi	$P\bar{3}m1$	-9.006	99.736	0.000	149.765	0.000	179.757	0.000	201.160	0.000	217.801	0.000
In_5Bi_3	$I4/mcm$	-24.235	273.574	0.000	407.247	0.000	487.274	0.000	544.370	0.000	588.753	0.000
Li_2InBi	$Fm\bar{3}m$	-11.734	72.900	0.000	128.089	0.000	165.373	0.000	192.911	0.000	214.625	0.000

Supplementary Notes

Supplementary note 1: High throughput thermal stability screening processing

Our stable material discovery procedure is described as following steps:

- Thermodynamical stability: high throughput DFT calculations are performed to screen thermodynamical stable compounds. The elements that are considered in our calculations are Li, Be, B, C, Na, Mg, Al, Si, K-As, Rb-Te, Cs, Ba, La, and Hf-Bi. For each composition X_2YZ, we considered three possible full Heusler structures. 70278 compounds are calculated and more than 1100 compounds are found to be stable (on the convex hull) and metastable (25 meV/atom above the convex hull).
- For all the thermodynamically stable and metastable compounds, we considered 15 different prototype structures for crystal structure determination. We found 593 compounds favor full Heusler structure.
- Phonon calculations are performed to filter out dynamically unstable compounds. 22 compounds are dynamically unstable.
- Electronic density of states calculations are performed to figure out the thermal and dynamically stable compounds are metal or semiconductors. 22 stable full Heusler compounds are semiconductors at PBE level.

Supplementary References

References

[1] Dshemuchadse, Julia and Steurer, Walter, More statistics on intermetallic compounds -ternary phases, Acta Crystallogr. A, 71, 335-345 (2015).

[2] Ran He et.al., Achieving high power factor and output power density in p-type half-Heuslers $Nb_{1-x}Ti_xFeSb$, Proc. Natl. Acad. Sci. USA, 113, 13576-13581 (2016).
